# Absence of Rybp Compromises Neural Differentiation of Embryonic Stem Cells

**DOI:** 10.1155/2016/4034620

**Published:** 2015-12-15

**Authors:** Gergo Kovacs, Viktoria Szabo, Melinda K. Pirity

**Affiliations:** Institute of Genetics, Laboratory of Embryonic and Induced Pluripotent Stem Cells, Biological Research Centre, Hungarian Academy of Sciences, Temesvári krt. 62, Szeged 6726, Hungary

## Abstract

Rybp (Ring1 and Yy1 Binding Protein) is a transcriptional regulator and member of the noncanonical polycomb repressive complex 1 with essential role in early embryonic development. We have previously described that alteration of Rybp dosage in mouse models induced striking neural tube defects (NTDs), exencephaly, and disorganized neurocortex. In this study we further investigated the role of Rybp in neural differentiation by utilising wild type (*rybp* ^+/+^) and *rybp null* mutant (*rybp* ^−/−^) embryonic stem cells (ESCs) and tried to uncover underlying molecular events that are responsible for the observed phenotypic changes. We found that *rybp null* mutant ESCs formed less matured neurons, astrocytes, and oligodendrocytes from existing progenitors than wild type cells. Furthermore, lack of *rybp* coincided with altered gene expression of key neural markers including Pax6 and Plagl1 pinpointing a possible transcriptional circuit among these genes.

## 1. Introduction

Complex network of transcription factors (TFs) control the gene expression in the developing embryo that manages complex differentiation processes. TFs also have important roles in the adult life in maintaining the pattern of differentiated gene expression and several of them function in multisubunit protein complexes [1]. Rybp (Ring1 and Yy1 Binding Protein, also known as Dedaf (Death Effector Domain-Associated Factor), Yeaf1 (Yy1 and E4tf1 Associated Factor 1); UniGene Mm.321633; MGI:1929059) is an evolutionarily conserved TF. It is also a member of the noncanonical mammalian polycomb repressive complex 1 (PRC1) [2, 3]. PRCs are important regulators of organogenesis and cell lineage specification because they are able to maintain pluripotency and repress differentiation. Rybp also acts as a bridging factor between E2f and Yy1 binding sites on target gene promoters, thus facilitating the formation of different multimeric TF complexes [4]. Complexes that form through these binding sites play important role in regulating cell proliferation and differentiation of multiple tissue lineages during early embryonic development. Rybp is also part of the BCOR complex (named after its BCL-6 corepressor subunit) [5], which plays important role in the differentiation of embryonic stem cells (ESCs) into ectoderm and mesoderm [6] and also is required for neurogenesis [7].

Our laboratory previously showed that Rybp is essential for early embryonic development, upregulated in certain cell types of the developing central nervous system (CNS), and that in a portion of the *rybp* ^+/−^ mice alterations in Rybp dosage resulted in striking neural tube defects (NTDs) and disorganization of the neocortex* in vivo *[8].

Here, we further characterized the role of Rybp in neural development. We utilized wild type (*rybp* ^+/+^) and* rybp null* mutant (*rybp* ^−/−^) ESCs, which lack functional Rybp protein, and differentiated them* in vitro* to neural lineages in order to reveal the function of Rybp in neural differentiation. Based on the* in vivo* evidences we hypothesized that in the absence of Rybp ESCs cannot undergo neural differentiation or have impaired neural differentiation ability. We showed impairment in neural lineage entry of ESCs in the lack of functional Rybp during* in vitro* neural differentiation. When analyzed in depth, the tumor suppressor Plagl1 (Pleomorphic Adenoma Gene-like 1 also known as Zac1 (Zinc Finger Protein Regulating Apoptosis and Cell-cycle Arrest) and Lot1 (Lost on Transformation 1) UniGene Mm.287857; MGI:1100874) was one of the most downregulated genes in the Rybp deficient cells. Since Plagl1 is a critical regulator of neural differentiation [9, 10] our results suggest that Plagl1 may, at least partially, mediate the effects of Rybp during neural differentiation.

## 2. Materials and Methods

### 2.1. Chemicals

All chemicals were purchased from Sigma-Aldrich (St. Louis, MO, USA), and culture media reagents were purchased from Invitrogen Life Technologies (Carlsbad, CA, USA), unless stated otherwise.

### 2.2. Cell Lines and Culture Condition

Mouse (129SV/Ola) R1 [11] (hereafter mentioned as* rybp* ^*+/+*^, control, or wild type) and D11 [8] (hereafter mentioned as *rybp* ^−/−^ or* null* mutant) (Figure S1 in Supplementary Material available online at http://dx.doi.org/10.1155/2016/4034620) ESCs were thawed on mitomycin C inactivated mouse embryonic fibroblast (MEF) layer and cultured on gelatin coated tissue culture plates as described [12]. The cells were maintained in ES medium: Dulbecco's Modified Eagle's medium (DMEM (1x) + GlutaMAX-I Dulbecco's Modified Eagle Medium, Gibco, REF 31966-021) contained 15% (vol/vol) fetal bovine serum (Gemini Stasis Stem Cell Qualified FBS, West Sacramento, CA, USA, Cat. Number 100-125), 0.1 mM nonessential amino acids (MEM Nonessential Amino Acids (100x), PAA, Cat. Number M11-003), 0.1 mM *β*-mercaptoethanol (2-Mercaptoethanol, Gibco, REF 31350-010), 50 U/mL penicillin/streptomycin (Penicillin/Streptomycin (100x), PAA, Cat. Number P11-010), and 1000 U/mL Leukemia Inhibitory Factor (LIF, ESGRO, Chemicon/Millipore, Billerica, MA, USA). The cells were passaged prior to reaching 70% confluency (approximately every 1-2 days). ESCs were cultured on gelatin coated dishes for at least two passages prior to differentiation to deplete potentially present MEF cells from the ESC culture. Cells were cultured in a humidified atmosphere containing 5% CO_2_ at 37°C. ES medium was changed daily.

### 2.3. Induction of Neural Differentiation by Retinoic Acid

**Figure 1:**
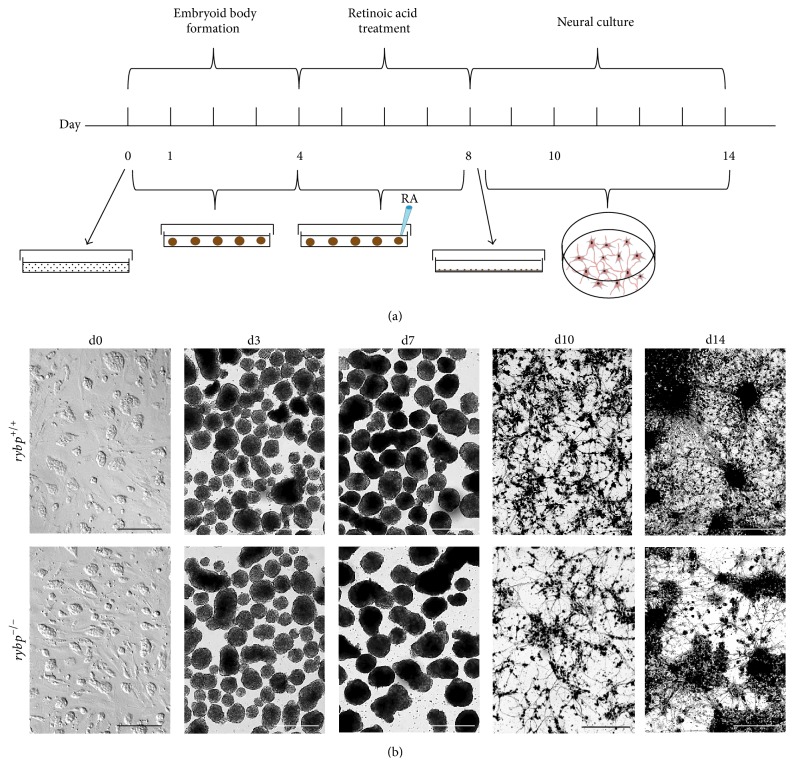
Morphological changes of *rybp* ^+/+^ and *rybp* ^−/−^ cells through* in vitro* neural differentiation. (a) Schematic illustration of* in vitro* neural differentiation as described previously by Bibel et al. (b) The *rybp* ^−/−^ ESCs display the same morphology in culture as the wild type (d0) and also form EBs (d3, d7). There is no noticeable difference between the two cell lines. After the plating (d10, d14) the *rybp* ^−/−^ cells grew less organized neural network than the wild type. Magnifications are 4x (d3, d7) and 10x (d0, d10, d14). Scale bars represent 500 *μ*m (d3, d7) and 200 *μ*m (d0, d10, d14).

ESCs were induced to differentiate into neuronal lineages as previously described, with some modifications (Figure 1(a)) [13]. In brief, mouse ESCs were harvested as single cell suspension using 0.05% (wt/vol) trypsin (Trypsin-EDTA (1x) 0.05%/0.02% in D-PBS, PAA, Cat. Number L15-004) and then seeded at a density of 4.5 × 10^5^ cells/mL in ES medium without LIF into 100 mm^2^ bacteriological dishes where cell attachment was prevented. ESCs were allowed to aggregate in suspension and form embryoid bodies (EBs) for 8 days. Medium was changed on every second day during differentiation unless stated otherwise. On the 4th day of EB formation 5 *μ*M all-trans retinoic acid (RA) (retinoic acid, Sigma, Cat. Number R2625-100MG) was added to the medium and EBs were cultured for a further 4 days in the presence of RA. Thereafter, 8-day-old EBs were dissociated using trypsin and plated onto poly-L-ornithine (Poly-L-Ornithine 0.01%, Sigma, Cat. Number P4957) and laminin (Laminin from Engelbreth-Holm-Swarm murine sarcoma basement membrane, Sigma, Cat. Number L2020-1MG) coated dishes at a density of 3 × 10^5^ cells/cm^2^ in DMEM/F-12 medium containing 3 mg/mL of D-(+)-glucose, 3 mg/mL AlbuMax I, 50 U/mL penicillin/streptomycin, and 1% (vol/vol) N-2 supplement (N-2 Supplement (100x), Gibco, Cat. Number 17502-048), and 10 ng/mL recombinant human bFGF (Fibroblast Growth Factor Basic, human, Sigma, Cat. Number F0291-25UG); the medium was changed 24 hours later. Two days after cells were plated, the medium was changed to DMEM/F12:Neuronal Base Medium (Neuronal Base Medium For Neuronal Cells, PAA, Cat. Number U15-023) (1 : 1), 1 mM GlutaMax, 3 mg/mL AlbuMax I, 50 U/mL penicillin/streptomycin, 0.5% (vol/vol) N-2 Supplement, and 1% (vol/vol) B-27 supplement (B-27 Supplement (50x), Gibco, Cat. Number 17504-044). The cells were harvested for further analyses on different time points of neural differentiation: days 0, 3, 7, 10, and 14 (labeled as d0, d3, d7, d10, and d14). On d10 and d14 the differentiated cells were also stained with cresyl violet in order to visualize the cell colonies and morphology of the cells. The images of the ESC cultures, EBs, and stained neural culture were obtained using Olympus cellR microscope (Olympus Corporation, Japan).

### 2.4. Quantitative Real-Time PCR (qRT-PCR)

For quantitation of mRNA levels during the* in vitro* differentiation quantitative real-time PCR (qRT-PCR) assays were performed. Total RNA was extracted from the harvested cells using Qiagen RNeasy Plus Mini Kit (Qiagen, Cat. Number 74134 Hilden, Germany) according to the manufacturer's instructions. cDNA synthesis was achieved with the isolated RNA using Applied Biosystems High capacity cDNA Reverse Transcription Kit (Invitrogen Life Technologies, Cat. Number 4368814 Carlsbad, CA, USA) according to the manufacturer's instructions.

qRT-PCR was performed in SYBR Green master mix (SYBR Select Master Mix for CFX, Applied Biosystems, REF 4472942) using Bioer LineGeneK Real-time PCR System (Bioer, China). Relative gene expression changes were quantified using the ΔΔCt method. The threshold cycle (Ct) values for each gene were normalized to expression level of Hprt, as internal control. To calculate fold change, the values were compared to undifferentiated samples (d0, *rybp* ^+/+^). The primers used in this study were listed in Supplementary Table 1.

### 2.5. Immunocytochemistry Analysis

For immunofluorescence staining of the cells, they were plated onto coverslips, differentiated into neuronal lineages as described above, and fixed with 4% (v/v) paraformaldehyde (PFA) for 20 min at room temperature (RT). After permeabilization in 0.2% Triton X-100 (Triton X-100, Sigma, Cat. Number T8787-250ML) in Phosphate Buffered Saline (PBS, DPBS (1x) Dulbecco's Phosphate Buffered Saline, Gibco, REF 14190-094) for 20 min at RT, cells were blocked in 5% Bovine Serum Albumin (BSA) (Albumin from bovine serum, Sigma, Cat. Number A-7906) in PBS for 1 h at RT and then sequentially incubated with the following primary antibody in 5% BSA overnight at 4°C: Nestin (Rat-401, Hybridoma Bank, Iowa, USA, 1 : 100), Pax6 (Pax6, Hybridoma Bank, Iowa, USA, 1 : 100), Tuj1 (Neuronal Class III *β*-Tubulin Polyclonal Antibody; Covance, Cat. Number PRB-435P, 1 : 2500), Gfap (Monoclonal Anti-Glial Fibrillary Acidic Protein Clone G-A-5, Sigma, Cat. Number G3893, 1 : 400), and Plagl1 (Zac1 (M-300), Santa Cruz Biotechnology, Cat. Number sc-22812, 1 : 1000). The cells were washed 5 times with PBS and incubated with fluorescent-labeled secondary antibodies (Alexa Fluor 647 Donkey-Anti-Mouse, Invitrogen, Cat. Number A-31571; Alexa Fluor 647 Goat-Anti-Rabbit, Invitrogen, Cat. Number A-21244; Alexa Fluor 488 Goat-Anti-Mouse, Invitrogen, Cat. Number A-10667) for 1 h at RT. Then the cells were washed 3 times with PBS. The cells were rinsed in 4′,6-diamidino-2-phenylindole (DAPI; Vector Laboratories, Cat. Number H-1200) for 20 min, washed 2 times with PBS, and mounted in 50% glycerol. The images were obtained using Olympus LSM confocal microscope (Olympus Corporation, Japan).

### 2.6. Statistical Analysis

All experiments were repeated three times. Experiments were evaluated with SPSS/PC+ version 17 program (SPSS, Chicago, IL) by using *t*-test type 3. Means are standard deviation. Values of *P* < 0.05 were accepted as significant (^*∗*^ *P* < 0.05; ^*∗∗*^ *P* < 0.01; ^*∗∗∗*^ *P* < 0.001).

## 3. Results

### 3.1. Neural Cell Culture Differentiated from *rybp* ^−/−^ ESCs Exhibit Different Morphology than Wild Type

In order to investigate the possible molecular mechanisms underlying the* in vivo* neural phenotypes of the *rybp* ^+/−^ mice we took advantage of the ESC based* in vitro* neural differentiation system. Thus, we adopted the protocol by Bibel et al. [13] in order to differentiate *rybp* ^+/+^ [11] and *rybp* ^−/−^ [8] ESCs lacking functional Rybp protein product towards neural cell lineages (Figure 1(a)). In brief, suspension cultures were initiated using 4.5 × 10^5^ cells/mL initial seeding density and were kept in suspension for 8 days to form EBs. RA was added to the suspension cultures on d4 and EBs were kept in RA supplemented medium until trypsinization on d8 and then seeded on poly-L-ornithine/laminin coated surfaces and differentiated further for another week. Samples for analysis were taken at d0, d3, d7, d10, and d14 and processed as required. *Rybp* ^−/−^ ESCs display similar morphology in culture as the control cell line (Figure 1(b) d0) and by d7 EBs grew larger compared to d3 in both cell lines (Figure 1(b), d3, d7) suggesting that *rybp* ^−/−^ cells are not compromised in their ability to form EBs and proliferate. As expected, after plating EBs on poly-L-ornithine and laminin coated surfaces, the wild type cells exhibited typical neuronal morphology and the cells grew axon-like outgrowth, connected to each other, and organized in a complex network consisting of heterogeneous cell population (Figure 1(b) d10, d14). However, *rybp* ^−/−^ cells grew less neurite processes and their organization into network was less extended in comparison to the wild type. This phenotype was independent from the initial cell density (Figure 1(b), d10, d14; data not shown). The gross morphology of the cells suggests that neuronal differentiation was affected by absence of* rybp*.

### 3.2. Expression of Pluripotency Markers in the Lack of Rybp during Neural Differentiation

**Figure 2:**
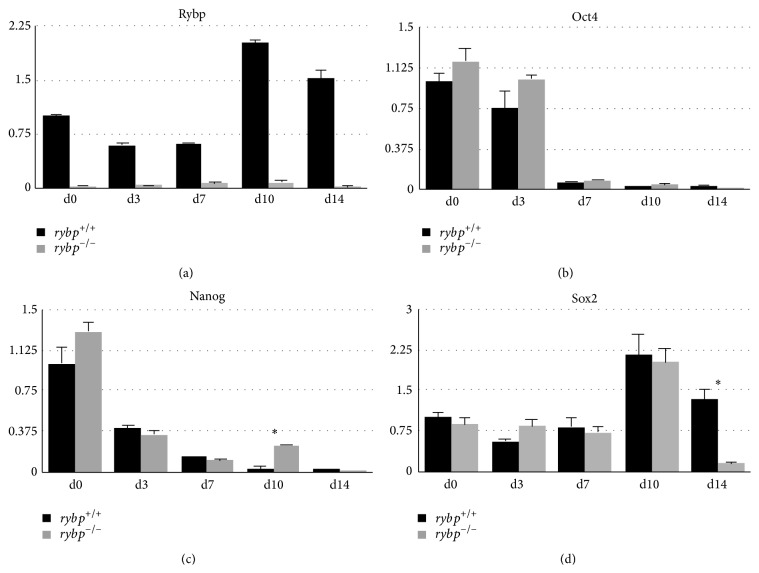
In the lack of Rybp there is no change in silencing of key pluripotency markers (Oct4, Nanog) during* in vitro* neural differentiation. Relative gene expressions of Rybp (a), Oct4 (b), Nanog (c), and Sox2 (d) were analyzed by qRT-PCR. For the analysis RNA was extracted and reverse-transcribed from differentiated neural cell culture generated from *rybp* ^+/+^ and *rybp* ^−/−^ ESCs. Samples are collected at days 0, 3, 7, 10, and 14 of differentiation. The expression of the indicated markers was normalized to Hprt expression, which was used as an internal control. Means are standard deviation. Values of *P* < 0.05 were accepted as significant (^*∗*^ *P* < 0.05; ^*∗∗*^ *P* < 0.01; ^*∗∗∗*^ *P* < 0.001). Statistical method: *t* test type 3.

To uncover underlying mRNA expression changes during* in vitro* neural differentiation we evaluated gene expression levels by qRT-PCR. First, we confirmed that the *rybp* ^−/−^ cells do not produce functional protein (Figure 2(a)). Then we analyzed the expression of pluripotency markers in both cell lines. Incomplete silencing of the pluripotency genes during differentiation inhibits neural differentiation. The analysis revealed that the expression of examined pluripotency markers Oct4 and Nanog was progressively downregulated in both cell lines by differentiation (Figures 2(b) and 2(c)) and there were no significant changes in the kinetics of pluripotency gene expression between the *rybp* ^+/+^ and *rybp* ^−/−^ cells.

Sox2 together with Oct4 and Nanog maintains pluripotency in stem cells and neural progenitors (NPCs) and, in addition, Sox2 is also important in the induction of neuroectoderm [14, 15]. The relative expression levels of Sox2 in both cell lines are similar with the exception of d14 when the level of Sox2 is diminished in the* rybp null* mutant whilst it stays high in the wild type (Figure 2(d)). This suggests that the differentiation ability of* rybp null* stem cells to NPCs or maintaining the characteristics of NPCs might be compromised. These results suggested that the silencing of key pluripotency genes exhibits similar kinetics with the exception of Sox2, which in the* rybp null* mutants drastically declines by d14.

### 3.3. Expression of Key Neural Markers in the Lack of Rybp during Neural Differentiation

After examination of the key pluripotency markers we analyzed the relative gene expression of key neural markers (e.g., Nestin, Pax6, NeuroD1, Tubb3, NeuN, Gfap, and Olig2) in both cell lines to gain information about the kinetics of neural differentiation.

**Figure 3:**
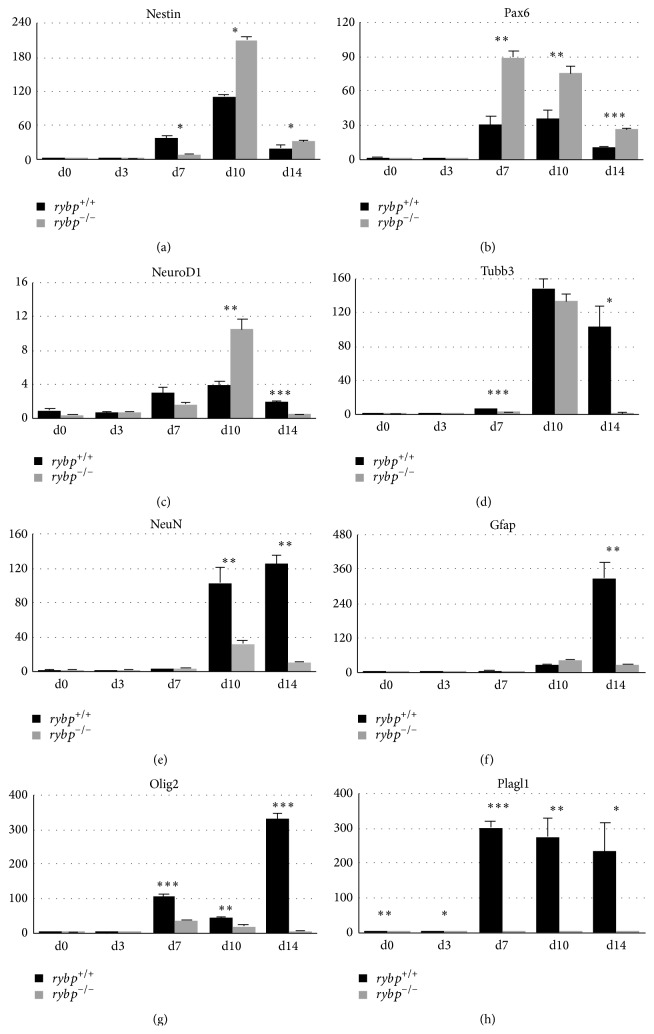
Neural markers (Nestin (a), Pax6 (b), NeuroD1 (c), Tubb3 (d), NeuN (e), Gfap (f), and Olig2 (g)) and transcription factor, Plagl1 (h), exhibit altered expression in the lack of Rybp during* in vitro* neural differentiation. Relative gene expression of key neural markers was analyzed with qRT-PCR. For the analysis RNA was extracted and reverse-transcribed from differentiated neural cell culture generated from *rybp* ^+/+^ and *rybp* ^−/−^ ESCs. Samples are collected at days 0, 3, 7, 10, and 14 of differentiation. The expressions of the indicated markers were normalized to Hprt expression, which was used as an internal control. Means are standard deviation. Values of *P* < 0.05 were accepted as significant (^*∗*^ *P* < 0.05; ^*∗∗*^ *P* < 0.01; ^*∗∗∗*^ *P* < 0.001). Statistical method: *t* test type 3.

**Figure 4:**
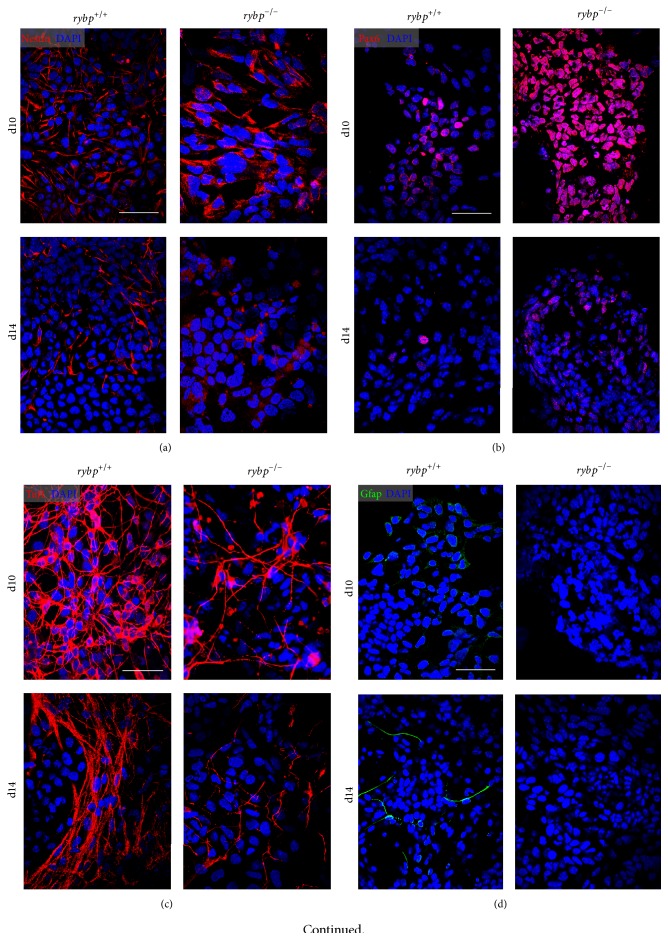
Rybp deficient cells show impaired protein expression of neural markers during* in vitro* neural differentiation. Immunocytochemical analysis of* in vitro* differentiated neural cultures shows that there are more Nestin (a) and Pax6 (b) and less Tuj1 (c) are visible in the* rybp null* mutant neural cell cultures by d10. Nestin and Tuj1 staining are reduced in the *rybp* ^−/−^ cell culture by d14 compared to wild type, which indicates possible defects in the late neural processes. Immunostaining of astrocyte marker, Gfap (d), shows a small amount of astrocyte-like cells in wild type culture, which is absent in the mutant. Immunocytochemistry with antibody against Plagl1 (e) reveals that Plagl1 is expressed in the nucleus of *rybp* ^+/+^ cells but it is absent in *rybp* ^−/−^ cells. Magnification is 60x. Scale bars represent 60 *μ*m.

Nestin is a widely employed marker of multipotent neural stem cells (NSCs) [16]. Once the cells become differentiated, Nestin expression is downregulated both* in vivo* and* in vitro* [17]. As expected, at the time of neural induction (d7, RA treatment) Nestin is strongly upregulated at d7 in both cell lines (Figure 3(a)). Nestin expression increased further after plating of the cells (d10) and its level decreased only at later phases of neural differentiation (d14) in both cell lines (Figure 3(a)). Notably, Nestin expression was more robust in the *rybp* ^−/−^ cell line compared to the *rybp* ^+/+^ at d10. Immunocytochemistry with Nestin antibody confirmed the presence of NPCs in both cell lines with stronger expression in the *rybp* ^−/−^ cells at d10 (Figure 4(a)). By the endpoint of* in vitro* neural differentiation (d14) the number of Nestin expressing cells was reduced in both cell lines (Figure 4(a); Figure S2(a) and (b)). These results suggest that early neural processes take place in a greater extent in the absence of functional Rybp.

Paired Box 6 (Pax6) gene is required for the initiation of neural differentiation and described as an essential factor for normal eye development as well [18]. Relative gene expression analysis revealed that Pax6 is upregulated after the RA treatment (Figure 3(b) from d7) and its level decreased at later time points of neural differentiation (d14) in the wild type cells. Expression of Pax6 was higher after neuroectodermal induction in the *rybp* ^−/−^ cells compared to the wild type (Figure 3(b) from d7). Pax6 immunostaining visualizes the distribution of Pax6 positive cells in the wild type and mutant neural cell cultures (Figure 4(b)). The Pax6 signal was stronger in the mutant cultures in all examined time points (Figure 4(b); Figure S2(c) and (d)). In both cell lines the number of Pax6 positive cells is higher at d10 when early differentiation events take place. These results correlate with the data of Pax6 qRT-PCR analysis and indicate an abundance of Pax6 positive cell population in the* rybp null* mutants.

Neurogenic Differentiation 1 (NeuroD1) is a TF, which is essential for terminal neuronal commitment of maturing neurons [19]. NeuroD1 drives premature differentiation of neuronal precursors into mature neurons. Relative gene expression analysis by qRT-PCR showed that the NeuroD1 is mildly expressed after RA treatment (d7) in both cell lines (Figure 3(c)). The expression level of NeuroD1 is significantly higher at early stage (d10) in the mutant cell line compared to the wild type. The expression of NeuroD1 decreased by the end of differentiation (d14) in the *rybp* ^−/−^ cells whilst there is only a minor decrease in its level in the *rybp* ^+/+^ cells. At d14, NeuroD1 expression is higher in the wild type cells compared to the mutants. This also confirms that the characteristics of NPCs are altered in the* rybp null* mutants: at the beginning/mid-phase of differentiation (d7 and 10) the premature differentiation of NPCs is accelerated but by the end of differentiation (d14) it is attenuated.

Class III Beta-Tubulin (Tubb3) plays a critical role in proper axon guidance, maintenance, and axonal transport [20, 21] and also is expressed in postmitotic phase of differentiating neurons [22]. Relative gene expression analysis by qRT-PCR showed that Tubb3 expression is elevated at early stage (d10) in both cell lines but its expression declines in the *rybp* ^−/−^ cells at later stage of neural differentiation (d14) while its level still remains high in the wild type (Figure 3(d)). In order to visualize ongoing neural differentiation spatiotemporally we stained the cells with Tuj1 antibody, which marks Tubb3 positive cells. The assessment of Tuj1 staining (Figure 4(c); Figure S2(e) and (f)) showed that neurite processes and axon-like structures start to develop in both cell lines however in the *rybp* ^+/+^ cells exhibit a complex and dense network of outgrowth compared to the mutants. Notably, Tubb3 also is expressed in the soma, around the nucleus in *rybp* ^+/+^ cells, while in the *rybp* ^−/−^ cells this staining was less visible. The reduced density of nerve fibers by ICC in the mutant cell line was consistent with the data on diminished amount of nerve bundles on Tubb3 by qRT-PCR analyses. These data suggest that a normal level of Rybp is important for the proper development of neurons.

In order to ascertain the presence of mature neurons in our cell cultures we assessed the relative gene expression changes of postmitotic neuronal marker NeuN (also known as Rbfox3). NeuN marks the nuclei of maturing and differentiated neurons [23]. As expected, qRT-PCR analysis showed that NeuN was abundantly expressed in the plated wild type neural cultures (d10, d14) but its level stayed constantly low in the mutants (Figure 3(e)). This indicates that much less mature neurons formed in the* rybp null* mutant culture (d10, d14) and further confirms the compromised differentiation ability of* rybp null* NPCs.

This observation tempted us to speculate whether this defect is specific for neurons or other cell types like astrocytes and oligodendrocytes are also affected in the absence of Rybp. Therefore we next analyzed whether cells lacking functional Rybp are able to generate astrocytes as well. In order to do so, we have performed relative gene expression analysis by qRT-PCR in both cell lines for the intermediate filament Glial Fibrillary Acidic Protein (Gfap), which is a commonly accepted marker of radial glia and astrocytes [24]. Our analysis revealed that the level of Gfap is elevated over ~300x in the wild type cells (Figure 3(f) d14). In comparison its level stayed low in the* rybp null* mutants (Figure 3(f) d14). We also analyzed the spatial distribution of Gfap by immunostaining and found that there were no Gfap positive cells in the mutant neural cultures (Figure 4(d); Figure S2(g) and (h)). This suggests that the astrocyte differentiation is defective in the mutant cells.

We also investigated if the absence of Rybp has influence on other types of glial cells or it is specific for astrocytes. For this purpose, we measured the relative expression level of Oligodendrocyte Lineage Transcription Factor 2 (Olig2) by qRT-PCR. Olig2 is a marker gene for oligodendrocytes [25–27]. Oligodendrocyte differentiation normally starts by d7 and peaks by d14 (Figure 3(g) *rybp* ^+/+^ cells). In contrast, the *rybp* ^−/−^ cells expressed 60x less Olig2 mRNAs at d14 compared to the wild type (Figure 3(g)). This demonstrates that oligodendrocyte differentiation is also impaired in the absence of Rybp.

### 3.4. Plagl1 Is a Downstream Target of Rybp during* In Vitro* Neural Differentiation

Previously, we have shown that Plagl1 is a candidate downstream gene of Rybp during* in vitro* cardiac differentiation of mouse embryonic stem cells [28]. Plagl1 has dual role; it is an important gene for cardiac development and it has also crucial role in neural development [10, 29]. Moreover, lack of Plagl1 in mouse models caused marked defects in CNS development (e.g., reduced size of cerebellum, reduced number of mature neurons) [9], which are similar to the phenotype of the *rybp* ^+/−^ mice (see above, Section 1) [8].

Therefore, next we have tested whether the differences in mRNA expression are also sustained (or changed) during neural differentiation between the wild type and* rybp null* mutant cell lines. Gene expression analysis (Figure 3(h)) revealed that Plagl1 is strongly induced (~300x) after the RA treatment (d7) during* in vitro* neural differentiation in the wild type cells and remained high until the end of differentiation (d14). However in the *rybp* ^−/−^ cells Plagl1 was not induced and showed complete deficiency.

After this, we wanted to test whether the difference in Plagl1 mRNA induction is also sustained at protein level. In order to visualize the spatiotemporal distribution of Plagl1 throughout the differentiation process we performed immunocytochemistry of* in vitro* neural cultures with antibody against the Plagl1 protein (Figure 4(e); Figure S2(i) and (j)). This confirmed that Plagl1 is visible in the nuclei of the wild type cells (Figure 4(e), wild type cells at d10 and d14) while in the *rybp* ^−/−^ cells there is a complete lack of Plagl1 staining. These results showed that Plagl1 induction is impaired in *rybp* ^−/−^ cells during the entire course of neural differentiation.

## 4. Discussion

Current work investigates the possible underlying molecular mechanisms responsible for the previously described phenotype of the* rybp* heterozygous mouse models [8]. Considerable attention has focused on the search for downstream target genes that may mediate the biological effect of Rybp in neural lineage commitment. Our study showed that the development of both neuronal and glial lineages is defective in the* rybp null* ESCs derivatives. Furthermore, our study establishes that the process of differentiation towards mature neural cell types rather than to a specific lineage is impaired in the lack of Rybp.

The contribution of *rybp* ^−/−^ ESCs to neural lineage commitment is particularly interesting since* in vivo* evidence has been already provided that Rybp is important for neural development and it is broadly expressed in different cell types of the CNS. Moreover misexpression of Rybp was shown in glioblastomas and other types of tumors [30].

**Figure 5:**
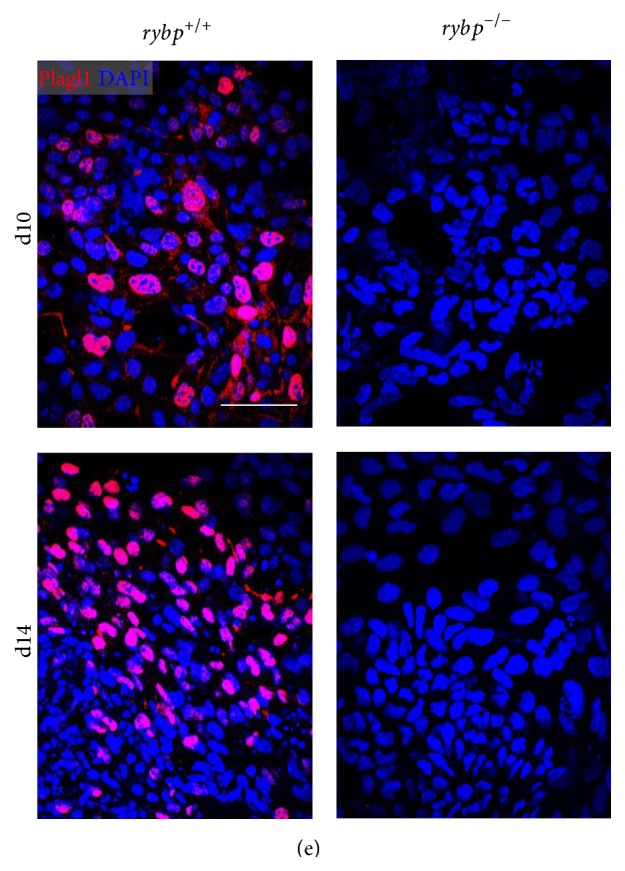
Model of* in vitro* neural differentiation in ESCs in presence and absence of Rybp. (a) Differentiation of wild type ESCs towards matured cell types of the CNS in the presence of Rybp. Pluripotent ESCs express key pluripotency markers (e.g., Oct4, Nanog) in undifferentiated state, which are gradually decreasing through* in vitro* neural differentiation. Parallel with this, early neural markers (Pax6, Nestin) are upregulated in NSCs, which will confer reduced proliferative and high differentiation capacity to the cells. On the other hand, in the presence of Rybp, upregulation of Plagl1 helps to facilitate late neural differentiation and inhibits cell proliferation, which will facilitate the generation of terminally differentiated neural cell types (N, OC, and AC). By the progression of neural differentiation, NPCs will differentiate further and start to express markers of specialized neural cell types (Tuj1, NeuN, Olig2, and Gfap). (b) Differentiation of pluripotent ESCs towards matured cell types of the CNS is impaired in the absence of Rybp. During neural differentiation early neural markers (Pax6, Nestin) exhibit elevated expression level in the* rybp null* mutants. These will facilitate the generation of a bigger pool of NPCs with higher proliferative capacity. On the other hand, in the absence of Rybp, Plagl1 expression is defective, which also contributes to increased proliferative and decreased differentiation capacity of the NPCs. As a result, the formation of terminally differentiated neural cell types (N, OC, and AC) will be impaired in the lack of functional Rybp. ESC: embryonic stem cell; NSC: neural stem cell; NPC: neural progenitor cell; N: neuron; OC: oligodendrocyte; AC: astrocyte; RA: retinoic acid; CNS: central nervous system.

Our results showed that the properties of* rybp null* NPCs are compromised:* rybp null* NPCs express Pax6, Nestin, and NeuroD1 in excess in comparison to the wild type ones. Pax6 is one of the key transcriptional factors [31], whose level needs to be upregulated at the beginning of neural development since Pax6 plays important role in establishing neuronal progenitor pool during the vertebrate nervous system development [32] (Figure 5). At the same time downregulation of Pax6 is also necessary for the forthcoming, late neural differentiation. In our assays when Rybp is absent, Pax6 is upregulated. Since high level of Pax6 obstructs terminal differentiation of NPCs it is possible that this elevated level of Pax6 helps to keep NPCs at the early stage of differentiation and inhibits the forthcoming late neural processes in the *rybp* ^−/−^ cells [33] (Figure 5). Upregulation of Nestin and NeuroD1 also confirms that early neural processes are accelerated in the lack of Rybp compared to the wild type. At the same time, silencing of key pluripotency genes (e.g., Oct4, Nanog) is a well coordinated event in the absence of Rybp indicating that improper silencing of pluripotency genes cannot be the causative of the phenotype. The only exception is Sox2 since the pool of Sox2 positive cells is reduced in the* rybp null* mutants earlier than in the wild type neural cultures. Sox2 is not only a critical factor for maintaining pluripotency and directing the differentiation of stem cells to neural progenitors but it is also important for maintaining the properties of NPCs [15]. Downregulation of Sox2 resulted in progenitor cells losing capability to both proliferate and terminally differentiate [34]; however to determine how Sox2 dose-change alters the state of NPCs in neural differentiation is a debate of future studies. Since others already described that striking increase of Pax6 partially rescues the Sox2 mutant phenotype [35], it cannot be excluded that increased level of Pax6 in the* null* mutant compensates the effect of low level of Sox2. It is possible that the lack of Rybp alters the balance among major transcription factors governing the NPC pool generation and consequent differentiation events. Changing the expression level of one or more members in these circuits will shift the finely tuned balance and initiate premature differentiation. Alternatively, this suboptimal balance may exhaust the NPC pool, which obscures consequent differentiation events, since Rybp is a moonlighting protein and is involved in such diverse biological functions as ubiquitination [3], apoptosis [36], and transcriptional repression [4], and it may have multiple roles in regulating neural differentiation as well. It further complicates the situation that Yaf2 (the other member of the Rybp/Yaf2 gene family) may compensate for the lack of Rybp at certain extent [37, 38].

Decreased level of late neuronal markers (Tuj1, NeuN, Gfap, and Olig2) indicates that maturing of neurons, oligodendrocytes, and astrocytes are deficient in the* rybp null* mutant cells (Figure 5). Parallel with these, the mutant cells seem to form a larger NPC pool (d10) and less astrocytes and oligodendrocytes in comparison to the wild types (d14). Overall, these observations confirm that maturation of neurons, astrocytes, and oligodendrocytes are deficient in the* rybp null* mutant cells and this process is not lineage specific.

The other gene whose expression is drastically changed in the *rybp* ^−/−^ neural cultures is Plagl1, which is an important regulator of neural development, apoptosis, and cell-cycle arrest whereas it has antiproliferative activity [39, 40]. Plagl1 is normally expressed in the progenitor cells of ventricular zone, subventricular zone, and external granular cell layer of the brain and promotes cell-cycle withdrawal of NPCs during the differentiation processes [10]. The tight regulation of cell-cycle processes is required to ensure timely generation and correct amount of the various neural cell types, which largely depends on the presence of Plagl1. The observation that Plagl1 is almost completely downregulated in the absence of functional Rybp and the fact that Rybp and Plagl1 deficient mice exhibit similar development defects (reduced number of mature neurons, reduced size of cerebellum, etc.) indicate a possible genetical or biochemical link between Rybp and Plagl1 [8, 9]. In our study increased level of Pax6 is accompanied by the downregulation of Plagl1 in Rybp deficient cells. In the absence of functional Rybp it is very likely that the balance of early and late neural processes is “unbalanced,” which is probably linked to the upregulation of Pax6 and/or the loss of Plagl1 (Figure 5).

Possibilities for action of Rybp are likely very diverse. Recent studies showed that Rybp is a member of the recently identified noncanonical PRC1 complex with unknown biological functions [41]. This complex is also encompassing Kdm2b, a gene with essential function during embryonic development. Notably, loss of Kdm2b is perinatally lethal with incomplete neural tube closure, exencephaly, and altered cell-cycle processes in NPCs [42] mimicking the phenotype of the* rybp* heterozygous mice [8]. Kdm2b, like Rybp, is also the member of the BCOR complex [5], which also plays important role in neurogenesis suggesting further possible functional cooperation between the two genes [7]. Importantly, members of the PRCs are proven to play important role in neural development: loss of Ring1B, which is core member of the canonical PRC1 and binding partner of Rybp, causes premature neuronal differentiation [43]. Ezh2, a core member of the PRC2, is highly expressed in NPCs and downregulated through the differentiation of mature cortical neurons [44]. Notably, Neurogenin 1, has been found to be suppressed by a PRC mediated mechanism at the neuronal-to-astrocytic transition of NPC differentiation during corticogenesis [45]. To clarify whether Rybp is functioning as the member of PRCs or other multimeric protein complexes during neural development is a debate of future studies.

## Supplementary Material

Supplementary Material contains a figure about the genomic structure of the mouse *rybp* gene in the wild type and the null mutant ESCs (Figure S1), the semi-quantitative analysis of the immunostaining (Figure S2) and a table which contains the list of primers used in qRT-PCR experiments (Table S1).
