# Supplementary material for: Absence of Rybp Compromises Neural Differentiation of Embryonic Stem Cells
Source: Stem Cells Int. 2015 Dec 15;2016:4034620. doi: 10.1155/2016/4034620 (PMC4693026; doi:10.1155/2016/4034620)
Supplement: Supplementary file 1 — Supplementary Material contains a figure about the genomic structure of the mouse rybp gene in the wild type and the null mutant ESCs (Figure S1), the semi-quantitative analysis of the immunostaining (Figure S2) and a table which contains the list of primers used in qRT-PCR experiments (Table S1). [file 4034620.f1.zip › Table S1.pdf]

Gene name	Forward primer sequence	Reverse primer sequence
Gfap	5’-AGATGAAACCAACCTGAGGC-3’	5’-CCTCCAGCGATTCAACCTTT-3’
Hprt	5’-AGTCCCAGCGTCGTGATTAG-3’	5’-GCAAGTCTTTCAGTCCTGTCC-3’
Nanog	5’-CAAGGGTCTGCTACTGAGATGCTCTG-3’	5’-TTTTGTTTGGGACTGGTAGAAGAATCAG-3’
Nestin	5’-AGTGCCCAGTTCTACTGGTGTCC-3’	5’-CCTCTAAAATAGAGTGGTGAGGGTTGA-3’
NeuN	5’-AATACATTTCGAGCTGCACCA-3’	5’-AACAAGCGTTTGCTCCAGT-3’
NeuroD1	5’-CGAGTCATGAGTGCCCAGCTTA-3’	5’-CCGGGAATAGTGAAACTGACGTG-3’
Oct4	5’-AGCCGACAACAATGAGAACC-3’	5’-TCTCCAGACTCCACCTCACA-3’
Olig2	5’-CGCAGCGAGCACCTCAAATCTAA-3’	5’-CCCAGGGATGATCTAAGCTCTCGAA-3’
Pax6	5’-CTTGGGAAATCCGAGACAGA-3’	5’-CTAGCCAGGTTGCGAAGAAC-3’
Plagl1	5’-ATGGCTCCATTCCGCTGTC-3’	5’-CTCAGCCTTCGAGCACTTGAA-3’
Rybp	5’-TTAGGAACAGCGCCGAAG-3’	5’-GCCACCAGCTGAGAATTGAT-3’
Sox2	5’-GCAGTACAACTCCATGACCA-3’	5’-TAGGACATGCTGTAGGTGGG-3’
Tubb3	5’-TGAGGCCTCCTCTCACAAGT-3’	5’-GGCCTGAATAGGTGTCCAAA-3’

Table 1 - List of primers used in this study
